# Navigating the Transition: Assessing Discharge Readiness in Persons With Severe Mental Illnesses in North India

**DOI:** 10.7759/cureus.117404

**Published:** 2026-10-03

**Authors:** Rashmita Saha, Garima Bharti, Anadrika Debbarma, Aarushi Aarushi, Nisha Prajapati, Pratibha Gehlawat

**Affiliations:** 1 Psychiatry, Institute of Human Behaviour and Allied Sciences, Delhi, IND; 2 Psychiatry, Employees' State Insurance Corporation (ESIC) Hospital, Basaidarapur, Delhi, IND

**Keywords:** discharge planning, discharge readiness, inpatient psychiatry services, schizophrenia, severe mental illness

## Abstract

Introduction

Severe mental illnesses (SMIs), including schizophrenia, bipolar disorder, and severe depressive disorders, contribute substantially to the burden of disease and mental health service utilisation in India. Despite this high burden and the critical role of safe care transitions, discharge readiness among psychiatric inpatients has not been systematically studied in India using validated patient-reported measures. This study assesses readiness for hospital discharge among inpatients with SMIs in a tertiary neuropsychiatric hospital in North India using the Readiness for Hospital Discharge Scale (RHDS).

Method

This cross-sectional study was conducted at Institute of Human Behaviour and Allied Sciences (IHBAS), a tertiary neuropsychiatric referral centre in Delhi, India. One-hundred one inpatients diagnosed with SMIs (schizophrenia, schizoaffective disorder, bipolar affective disorder, or severe depressive disorder) as per the International Classification of Diseases, 10th Revision, Diagnostic Criteria for Research (ICD-10 DCR) were recruited through purposive sampling over six months. Discharge readiness was assessed on the planned day of discharge using the Hindi-translated RHDS-Adult Form. Sociodemographic and clinical details were recorded using a semi-structured proforma. Associations were examined using bivariate analyses and logistic regression.

Results

Schizophrenia (47.5%) and bipolar disorder (28.7%) were the most common diagnoses. The mean RHDS score was 7.07 ± 1.08, indicating upper-moderate readiness. Domain scores were highest for coping ability and expected support, intermediate for personal status, and lowest for knowledge. Schizophrenia was significantly associated with lower readiness (p = 0.002) and independently predicted poor discharge readiness (adjusted odds ratio (OR) = 0.13, 95% CI 0.04-0.46). Longer length of stay predicted higher readiness (OR = 1.07/day, p = 0.032).

Conclusion

Despite overall clinical stability at discharge, psychiatric inpatients demonstrated suboptimal readiness, particularly in the knowledge domain, indicating inadequate understanding of illness, treatment, relapse prevention, and follow-up care. The findings underscore the need for structured and standardized discharge planning with strengthened psychoeducation, clear information on community resources and legal rights, and greater involvement of multidisciplinary teams such as social workers or case managers. Tailored discharge interventions may contribute to continuity of care and post-discharge outcomes. Nevertheless, prospective Indian studies are needed to validate its effectiveness and the extent of these benefits.

## Introduction

Severe mental illnesses (SMIs), such as schizophrenia, bipolar disorder, and severe depressive disorders, substantially contribute to the overall burden of disease and service utilisation in India. The National Mental Health Survey (NMHS) 2015-2016 estimated a point prevalence of any mental morbidity of 10.6%, with a lifetime prevalence of 13.7% [1,2]. The study also showed a glaring treatment gap, ranging from 70% to 92% across various psychiatric illnesses, including a gap of 74% for severe mental disorders [1,2]. These findings reflect enduring obstacles in awareness, accessibility, affordability, and continuity of care, underscoring the need to strengthen the entire mental healthcare pathway, from acute hospital-based services to community reintegration.

In the Indian context, inpatient psychiatric care continues to play a pivotal role, especially for clinical scenarios such as acute symptom exacerbation, high-risk suicidal behaviour, severe agitation or aggression, non-adherence, and co-occurring medical or psychiatric complications. Data from tertiary centres indicate median lengths of stay (LOS) typically between two and four weeks (e.g., 14-18 days), with longer stays for psychotic and bipolar disorders [3,4]. General Hospital Psychiatry Units (GHPUs) have further accelerated a national shift towards shorter, more efficient admissions with higher patient turnover. This trend is supported by advances in pharmacotherapy, including the wider availability of long-acting injectables, and the gradual expansion of community-oriented models of care aimed at reducing long-term institutionalisation [4].

The need for optimised inpatient care and well-coordinated discharge processes is compounded by the economic and human-resource constraints that shape mental healthcare delivery in India. Analyses of National Sample Survey data suggest substantial household spending, roughly ₹26,800 for average neuropsychiatric admissions [5]. Moreover, human resources remain a binding constraint. The World Health Organization’s Mental Health Atlas series has reported on low psychiatrist density in India (approximately 0.75 psychiatrists per 100,000 population), while Indian experts have argued for a desirable threshold of ≥3 psychiatrists per 100,000 as a realistic goal [6]. Given the magnitude of the resource deficit, it becomes imperative to ensure that each inpatient episode is utilised effectively and that transitions out of the hospital minimise relapse risk and reduce preventable 'revolving-door' readmissions.

In this landscape, discharge planning emerges as not merely an administrative formality but a crucial clinical process, intimately linked with patient safety, functional recovery, continuity of care, and system efficiency. Evaluating whether patients feel prepared and confident to leave the hospital is therefore central to quality improvement in psychiatric services. Readiness for hospital discharge refers to the extent to which a patient is prepared to continue their recovery safely after leaving the hospital. It serves as an indicator of whether a patient can be discharged without undue risk. One of the most widely used and validated instruments to measure this construct internationally is the Readiness for Hospital Discharge Scale (RHDS) [7].

International research demonstrates that patient-reported readiness for discharge predicts post-discharge complications, emergency visits, and readmissions [8]. In India, research on discharge readiness, particularly using structured tools such as the RHDS, is limited, especially in psychiatric populations. In the absence of robust Indian studies, international literature provides important insights into discharge readiness. Studies in obstetric populations, particularly among postnatal mothers, have shown that higher discharge readiness is associated with better self-care competence, reduced anxiety, and improved early post-discharge outcomes, whereas inadequate readiness reflects insufficient discharge counselling and poor understanding of home care needs [9,10]. Similarly, research in chronic medical conditions, including heart failure and chronic obstructive pulmonary disease (COPD), has demonstrated that structured discharge education improves readiness, adherence, and follow-up, while low readiness is associated with increased unplanned healthcare utilisation [11-13]. These findings underscore the importance of systematic discharge planning and highlight the need for further research in Indian psychiatric populations.

However, despite the high burden of SMIs and the central importance of safe transitions in psychiatric care, to the best of our knowledge, no published Indian study has systematically assessed discharge readiness among psychiatric inpatients using a validated, patient-reported measure such as the RHDS. This study examines the readiness for hospital discharge among psychiatric inpatients suffering from SMIs in a tertiary neuropsychiatric hospital in North India using the RHDS [7]. By foregrounding a patient-reported outcome at the critical point of hospital-to-community transition, the study aims to inform practical, scalable strategies for discharge planning that can enhance recovery trajectories, reduce avoidable readmissions, and improve the utilisation of India’s limited psychiatric inpatient resources.

## Materials and methods

Study design, setting, and participants

The study utilised a cross-sectional design. It was conducted at the Institute of Human Behaviour and Allied Sciences (IHBAS), a tertiary-care hospital located in Delhi, India, which functions as a specialised referral centre for neuropsychiatric disorders and caters to a large population from Delhi and adjoining northern states. We included adult inpatients diagnosed with SMIs, namely, schizophrenia, schizoaffective disorder, bipolar affective disorder, and severe depressive disorder, as per the International Classification of Diseases, 10th Revision, Diagnostic Criteria for Research (ICD-10 DCR) [14]. Patients were excluded if they or their nominated representative refused consent or if they had a critical medical illness or severe cognitive impairment that precluded assessment of discharge readiness. Purposive sampling over a six-month period was employed to ensure inclusion of clinically stable inpatients who were planned for discharge during the study period. During the study period, 115 patients were approached, of whom 109 met the eligibility criteria. Eight eligible patients were subsequently excluded, resulting in a final sample of 101 inpatients. This sampling strategy enabled assessment of discharge readiness at the clinically relevant transition point preceding discharge but may have introduced selection bias by underrepresenting patients who were acutely unwell, clinically unstable, or not considered ready for discharge. The study was approved by the Institutional Ethics Committee of IHBAS. Written informed consent was obtained from all participants or their legally authorized representatives. Participants were assured of confidentiality, the voluntary nature of participation, and their right to withdraw at any stage without any impact on their treatment.

Instruments

Data were collected on the day of planned discharge by a trained study investigator familiar with the study instruments. Although formal blinding was not feasible due to the study design, efforts were made to standardise data collection and minimise interviewer bias by having the same investigator administer the instruments. A semi-structured proforma was used to record the participants’ socio-demographic details (age, gender, education, socioeconomic status, etc.), clinical profile (diagnosis, length of stay, use of long-acting injections or somatic therapy like electroconvulsive therapy), caregiver-related information, and discharge preparation (including details of discharge education and discussions on medication, follow-up, and relapse prevention). The participants were assessed for their readiness for hospital discharge on the RHDS - Adult Form [7] and severity of their clinical condition on the Global Assessment of Functioning (GAF) scale [15]. A detailed description of these two instruments is given below.

Readiness for Hospital Discharge Scale - Adult Form (RHDS)

The RHDS is a patient-rated instrument originally developed by Weiss and colleagues to evaluate perceived readiness for discharge across four domains: Personal Status - physical and emotional well-being, Knowledge - understanding of the care plan and post-discharge needs, Coping Ability - self-confidence in managing recovery, and Expected Support - availability of help after discharge [7]. The adult version comprises 21 items, each rated on an 11-point Likert scale (0 = not at all ready to 10 = totally ready). Higher scores indicate greater readiness for discharge. The total RHDS score is calculated as the mean of all item scores, with higher values reflecting better discharge readiness. For analysis, a score of <7 was considered indicative of low discharge readiness, consistent with the cutoff used in previous studies employing the 21-item RHDS. [16] The scale has demonstrated good reliability (Cronbach’s α > 0.85) and construct validity across multiple clinical populations [7]. 

Given that the majority of the patient population at our institute is Hindi-speaking, the RHDS was translated into Hindi in accordance with WHO’s standard protocol for translation and adaptation of instruments, which included forward translation, expert panel review, back-translation, pre-testing, and cognitive interviewing to ensure semantic and conceptual equivalence. Permission for use and translation of the instrument was obtained from the original author. However, formal psychometric validation (e.g., reliability and construct validity) was not conducted and represented a limitation of this study.

Permission to use the RHDS and translation to Hindi version using WHO guidelines was obtained from the original author (Weiss, Marianne E., "Readiness for Hospital Discharge Scale - Adult Form - English" (2006)) (see Appendix for the Hindi and English versions of the RHDS.

Global Assessment of Functioning (GAF) Scale

The GAF is a clinician-rated scale designed to assess an individual’s overall level of psychological, social, and occupational functioning. It was included as Axis V in the multiaxial system of the Diagnostic and Statistical Manual of Mental Disorders, Fourth Edition published by the American Psychiatric Association. It is free to use for research and does not require permission from the American Psychiatric Association. The GAF is scored on a continuum from 0 to 100, with higher scores indicating better functioning. The scale provides anchor points at 10-point intervals that describe symptom severity and functional impairment. Ratings are based on the clinician’s judgment of the individual’s functioning at the time of assessment. Studies have shown that the GAF demonstrates moderate reliability, though inter-rater variability can occur due to subjective clinical judgment [15].

Data analysis

Data were analysed using IBM SPSS Statistics (version 21.0, IBM Corp., Armonk, NY). Descriptive statistics (means, standard deviations, frequencies, and percentages) were computed for sociodemographic and clinical variables. RHDS scores were summarised as means and standard deviations. Inferential statistics (t-tests, ANOVA, or non-parametric equivalents) were applied to examine associations between discharge readiness scores and patient characteristics. A p-value <0.05 was considered statistically significant.

## Results

The mean RHDS score was 7.07 (±1.08). Composite means for the four domains indicated good personal status (7.17 ± 0.91), strong coping (7.56 ± 1.40), expected support (7.42 ± 1.61) and intermediate knowledge (6.83 ± 1.54). Among the 101 participants, 47 patients had low readiness (RHD < 7), and 54 had high readiness (RHD ≥ 7). The sociodemographic and clinical characteristics of patients stratified by readiness for hospital discharge are presented in Table 1. Categorical variables, e.g., sex, education, socioeconomic status etc., were compared using Pearson's Chi-square test and continuous variables, like age, duration of hospital stay and GAF scores, were assessed using an independent-samples t-test. There was no significant difference between the two groups in terms of sex distribution, with males comprising the majority in both groups (78.7% in the low-readiness group and 79.6% in the high-readiness group; p = 0.911). Educational status was also comparable across groups (p = 0.520), with high school and senior secondary/diploma qualifications being the most common levels attained. Socioeconomic status did not differ significantly between groups (p = 0.153), although a larger proportion of patients in the low-readiness group belonged to the lower socioeconomic strata. 

**Table 1:** Sociodemographic and clinical profile of patients with SMI Abbreviation: RHD = Readiness for Hospital Discharge, LAI = long-acting injection, ECT = electroconvulsive therapy, LMSES = lower-middle socioeconomic status, LES = lower socioeconomic status, UMSES = upper-middle socioeconomic status, GAF = Global Assessment of Functioning scale * p-value < 0.05 considered significant.

Variable	Category	Low readiness (RHD < 7) n (%) (n = 47)	High readiness (RHD ≥ 7) n (%) (n = 54)	Test statistics	df	p-value	Effect size
Sex	Female	10 (21.3)	11 (20.4)	χ²=0.013	1	0.911	0.011
	Male	37 (78.7)	43 (79.6)				
Education level	Graduate	6 (12.8)	13 (24.1)	χ²≈4.21	5	0.520	0.203
	High school	16 (34.0)	15 (27.8)				
	Illiterate	4 (8.5)	4 (7.4)				
	Postgraduate	1 (2.1)	2 (3.7)				
	Primary	10 (21.3)	6 (11.1)				
	Senior secondary / Diploma	10 (21.3)	14 (25.9)				
Diagnosis (ICD)	Schizophrenia (F20)	31 (66.0)	18 (33.3)	χ²≈14.61	3	0.002*	0.362
	Schizoaffective (F25)	0 (0.0)	3 (5.6)				
	Bipolar affective disorder (F31)	10 (21.3)	26 (48.1)				
	Depressive episode (F32)	6 (12.8)	7 (13.0)				
LAI / modified ECT	Yes	22 (46.8)	28 (51.9)	χ²=0.256	1	0.613	0.050
	No	25 (53.2)	26 (48.1)				
Socioeconomic status	LMSES	4 (8.5)	2 (3.7)	χ²≈5.26	3	0.153	0.227
	LSES	23 (48.9)	17 (31.5)				
	UMSES	11 (23.4)	20 (37.0)				
	USES	9 (19.1)	15 (27.8)				
Age (years)	Mean (±SD)	33.57 (10.29)	35.30 (11.77)	-0.78	99	0.439	0.16
Severity (GAF score)	Mean (±SD)	47.45 (16.32)	45.13 (16.23)	0.72	99	0.477	0.14
Duration of hospital stay (days)	Mean (±SD)	22.02 (10.13)	26.59 (11.50)	-2.11	99	0.039*	0.42

A statistically significant association was observed between psychiatric diagnosis and readiness for discharge (p = 0.002). Patients with schizophrenia (F20) constituted a substantially higher proportion of the low-readiness group (66.0%) compared to the high-readiness group (33.3%). By contrast, bipolar affective disorder (F31) was more common among patients with high readiness (48.1%) than those with low readiness (21.3%). Schizoaffective disorder (F25) was present only in the high-readiness group, though in small numbers, while depressive episodes (F32) were similarly distributed across both groups.

There were no significant differences between groups with respect to receipt of long-acting injectables (LAIs) or modified ECT during admission (p = 0.613). Mean age and severity of illness as measured by GAF scores were also comparable between the two groups (p = 0.439 and p = 0.477, respectively). However, duration of hospital stay differed significantly, with patients in the high-readiness group having a longer mean length of stay (26.59 ± 11.50 days) compared to those in the low-readiness group (22.02 ± 10.13 days; p = 0.039).

Overall, as shown in Table 1, psychiatric diagnosis and length of hospital stay emerged as significant factors associated with readiness for discharge, whereas sociodemographic variables, illness severity, and treatment modalities did not demonstrate statistically significant differences between groups.

The associations between discharge planning variables, information provided at discharge, and readiness for hospital discharge were assessed using the chi-square test and are presented in Table 2. Notification of discharge was common in both groups (89.4% in the low-readiness group vs. 79.6% in the high-readiness group), with no statistically significant difference (p = 0.182). Similarly, initiation of discharge planning did not differ between groups (42.6% in low readiness vs. 40.7% in high readiness; p = 0.854). The number of members involved in discharge planning was also comparable (p = 0.182), with most cases involving a single member in both groups. 

**Table 2:** Details of discharge planning for patients with SMI Abbreviations: RHD = Readiness for Hospital Discharge, MHE = Mental Health Establishment, SMI = severe mental illness *p value <0.05 considered significant

Variable		Category	Low readiness (RHD < 7) n (%) (n = 47)	High readiness (RHD ≥ 7) n (%) (n = 54)	χ²	df	p-value	Effect size
Notification of discharge		Yes	42 (89.4)	43 (79.6)	1.785	1	0.182	0.133
		No	5 (10.6)	11 (20.4)				
Initiation of discharge planning		Yes	20 (42.6)	22 (40.7)	0.034	1	0.854	0.018
		No	27 (57.4)	32 (59.3)				
Members included in discharge planning		0	0 (0.0)	2 (3.7)	4.868	3	0.182	0.192
		1	38 (80.9)	45 (83.3)				
		2	9 (19.1)	6 (11.1)				
		3	0 (0.0)	1 (1.9)				
Information provided at the time of discharge	Signs of illness	Yes	17 (36.2)	14 (25.9)	1.24	1	0.266	0.111
		No	30 (63.8)	40 (74.1)				
	Coping strategies	Yes	13 (27.7)	15 (27.8)	0.00	1	0.989	0.001
		No	34 (72.3)	39 (72.2)				
	Warning signs	Yes	20 (42.6)	17 (31.5)	1.327	1	0.249	0.115
		No	27 (57.4)	37 (68.5)				
	Emergency help	Yes	28 (59.6)	26 (48.1)	1.319	1	0.251	0.114
		No	19 (40.4)	28 (51.9)				
	Current treatment	Yes	15 (31.9)	17 (31.5)	0.002	1	0.963	0.005
		No	32 (68.1)	37 (68.5)				
	Medication side effects	Yes	14 (29.8)	18 (33.3)	0.146	1	0.702	0.038
		No	33 (70.2)	36 (66.7)				
	Non-pharmacological interventions	Yes	9 (19.1)	10 (18.5)	0.007	1	0.936	0.008
		No	38 (80.9)	44 (81.5)				
	Nearest MHE	Yes	7 (14.9)	5 (9.3)	0.762	1	0.383	0.087
		No	40 (85.1)	49 (90.7)				
	Legal rights	Yes	6 (12.8)	3 (5.6)	1.610	1	0.297	0.126
		No	41 (87.2)	51 (94.4)				
	Post-discharge follow-up	Yes	25 (53.2)	41 (75.9)	5.735	1	0.017*	0.238

With regard to specific discharge information provided, no significant differences were observed between low- and high-readiness groups for education on signs of illness (p = 0.266), coping strategies (p = 0.989), warning signs (p = 0.249), emergency help (p = 0.251), current treatment (p = 0.963), medication side effects (p = 0.702), non-pharmacological interventions (p = 0.936), nearest mental health establishment (p = 0.383), or legal rights (p = 0.297). However, provision of post-discharge follow-up instructions was significantly associated with higher readiness (p = 0.017). A substantially greater proportion of patients in the high-readiness group (75.9%) received follow-up instructions compared to those in the low-readiness group (53.2%). Overall, as shown in Table 2, structured informational components of discharge were generally sparse and largely non-significant, except for follow-up planning, which demonstrated a meaningful association with discharge readiness.

The results of the logistic regression analysis are summarised in Table 3. Diagnosis of schizophrenia (F20) emerged as a strong and statistically significant negative predictor of high readiness for discharge (OR = 0.13, 95% CI: 0.04-0.46, p = 0.002), indicating that patients with schizophrenia were significantly less likely to report high readiness compared to other diagnostic groups. In contrast, length of hospital stay was a positive predictor of readiness, with each additional day of stay increasing the odds of high readiness by approximately 7% (OR = 1.07, 95% CI: 1.01-1.13, p = 0.032). However, given the cross-sectional design, this association should not be interpreted as evidence that a longer hospital stay itself improves discharge readiness. Other variables, including age, gender, education level, socio-economic status, and diagnoses other than schizophrenia, were not statistically significant predictors in the adjusted model (all p > 0.1). Thus, Table 3 highlights that diagnostic category and duration of inpatient care were the primary independent determinants of discharge readiness. 

**Table 3:** Results of the logistic regression analysis Abbreviation: LAI = long-acting injection, ECT = electroconvulsive therapy, LMSES = lower-middle socioeconomic status, LES = lower socioeconomic status, UMSES = upper-middle socioeconomic status, GAF = Global Assessment of Functioning scale, SMI = severe mental illness *p < 0.05 considered significant

Variable	Univariable OR (95% CI)	p-value	Adjusted OR (95% CI)	p-value
Age	1.008 (0.972–1.044)	0.673	1.002 (0.956–1.051)	0.931
Female vs male	0.856 (0.326–2.242)	0.751	1.747 (0.507–6.025)	0.377
Education				
High school vs. graduate	0.560 (0.169–1.858)	0.344	0.667 (0.156–2.854)	0.585
Illiterate vs. graduate	0.462 (0.085–2.502)	0.370	0.588 (0.076–4.525)	0.610
Postgraduate vs. graduate	0.923(0.069–12.280)	0.952	0.388 (0.017–8.723)	0.551
Primary vs. graduate	0.277 (0.068–1.123)	0.072	0.365 (0.071–1.863)	0.226
Senior secondary/diploma vs. graduate	0.646 (0.183–2.284)	0.498	0.775 (0.165–3.652)	0.748
Schizophrenia (F20) vs. other SMIs	0.257 (0.112–0.589)	0.001	0.13 (0.04-0.46)	0.002*
GAF score	0.991 (0.967–1.015)	0.453	1.006 (0.974–1.040)	0.719
Duration of hospital stay (days)	1.042 (1.000–1.085)	0.048	1.07 (1.01-1.133)	0.032*
LAI/modified ECT: Yes vs. No	1.228 (0.559–2.694)	0.609	1.240 (0.441–3.482)	0.684
Socioeconomic status				
LMSES vs. LSES	0.553 (0.091–3.368)	0.520	0.714 (0.095–5.391)	0.744
UMSES vs. LSES	2.211 (0.829–5.893)	0.113	2.173 (0.680–6.950)	0.191
USES vs. LSES	1.658 (0.602–4.564)	0.328	1.448 (0.402–5.221)	0.571

The comparison of readiness for hospital discharge between schizophrenia and other SMIs is presented in Table 4. Patients with schizophrenia (F20) had the lowest mean readiness score (mean = 6.47 ± 1.08), indicating comparatively poorer discharge preparedness. By contrast, patients with other SMIs, including schizoaffective disorder (F25), bipolar affective disorder (F31), and depressive disorder (F32), demonstrated a higher composite mean readiness score (mean = 7.29 ± 1.02), reflecting better overall preparedness for discharge. The mean difference between the groups was 0.82 (95% CI: 0.40-1.24). An independent-samples t-test demonstrated that this difference was statistically significant (t(98) = 3.92, p < 0.001). The Cohen’s d of 0.78 indicates a moderate-to-large effect size, suggesting that diagnostic category was associated with a meaningful difference in RHD scores, with participants with other SMIs showing higher RHD scores than those with schizophrenia. As depicted in Table 4, other SMIs were associated with relatively greater discharge readiness compared to schizophrenia, underscoring the need for diagnosis-specific discharge planning strategies. 

**Table 4:** Comparison of readiness for hospital discharge between schizophrenia and other SMIs The other SMI group includes mood-related disorders (F25, F31, F32). Abbreviation: RHD = Readiness for Hospital Discharge, SMI = severe mental illness *p < 0.05 considered significant

Diagnostic category	n	Mean RHD ± SD	Mean difference (95% CI)	t (df)	p-value	Cohen's d
Schizophrenia	49	6.47 ± 1.08	0.82 (0.40–1.24)	3.92 (98)	<0.001*	0.78
Other SMIs	52	7.29 ± 1.02				

## Discussion

The present study assessed readiness for hospital discharge among psychiatric inpatients diagnosed with severe mental illnesses in a tertiary neuropsychiatric centre. The findings highlight important diagnostic differences, gaps in discharge preparation, and factors influencing perceived readiness.

Overall, patients reported a moderate to high level of readiness, with higher scores in the domains of Coping Ability, Expected Support, and Personal Status. This suggests that most patients perceived themselves as emotionally stable at discharge and anticipated adequate family support - an observation consistent with the Indian context, where caregivers play a central role in treatment continuation. However, the Knowledge domain received the lowest scores, indicating that patients felt less confident about their illness, medications, early warning signs, and follow-up care. This reflects a critical gap in discharge education and is consistent with previous studies reporting inadequate informational preparedness among psychiatric inpatients [17].

A key finding was the significant association between psychiatric diagnosis and readiness for discharge. Patients with schizophrenia demonstrated the lowest readiness, both in bivariate and multivariate analyses. This is in line with literature suggesting that cognitive deficits, limited insight, and impaired social functioning frequently hinder self-management abilities in schizophrenia [18]. By contrast, patients with other SMIs (bipolar disorder, schizoaffective disorder, depressive disorder) displayed higher readiness scores, possibly due to better recovery of insight and cognitive clarity by the time of discharge [19]. The comparison presented in Table 4 further supports these diagnostic differences, clearly showing lower readiness among non-affective psychotic disorders compared to affective disorders. 

Another important observation was the positive association between length of hospital stay and readiness. A longer stay may provide additional time for clinical stabilisation, caregiver discussions, and psychoeducation, thereby improving preparedness. This association should be interpreted cautiously, as it may also be influenced by confounding factors such as clinician judgment regarding discharge timing, illness recovery trajectory, level of insight, and other unmeasured clinical and psychosocial factors. Although some earlier studies associate prolonged hospitalisation with illness severity and poorer outcomes, the present findings suggest that, in this setting, adequate inpatient duration may contribute positively to readiness [19]. The observed differences from previous studies may also reflect variations in healthcare systems, discharge planning practices, and cultural contexts. 

Discharge communication practices revealed notable weaknesses. Although the majority of patients were notified of discharge, only a minority received structured discharge planning, and most information was delivered orally. Written instructions were rarely provided, and essential components, such as medication side effects, non-pharmacological strategies, relapse warning signs, and legal rights, were inconsistently discussed. These gaps raise concerns about continuity of care, adherence, and relapse prevention. Importantly, patients who received post-discharge follow-up instructions showed significantly higher readiness, highlighting the value of even simple, structured guidance at discharge.

Schizophrenia remained a strong negative predictor even after adjusting for other variables. Factors such as age, gender, education, baseline severity, and general discharge planning steps did not significantly influence readiness, indicating that clinical characteristics may be more relevant than sociodemographic factors in this context. The findings reinforce the importance of diagnosis-specific discharge planning, particularly for patients with schizophrenia. This may include repeated and simplified teaching, caregiver training, written plans, crisis instructions, medication reconciliation, and early post-discharge follow-up. The RHDS scale can be integrated into routine discharge procedures as a quick screening tool to flag patients with inadequate readiness. Patients scoring below 7 may benefit from additional interventions to ensure safety and continuity of care.

Comparisons with previous literature show consistency in key observations, including moderate readiness levels and lower knowledge domain scores [19,20]. However, unlike many earlier studies focusing on single diagnostic groups, the present study examines multiple psychiatric diagnoses, allowing clearer differentiation of diagnostic influences on discharge preparedness.

This study has several limitations. It was conducted at a single tertiary-care neuropsychiatric centre, which may limit the generalizability of the findings to other settings. The sample size was modest, and purposive sampling may have introduced selection bias, potentially affecting representativeness. The cross-sectional design precludes causal inference between discharge readiness and associated factors. Although the Readiness for Hospital Discharge Scale (RHDS) was translated into Hindi using standard WHO procedures, formal psychometric validation in this sample was not performed as the primary objective was to assess discharge readiness and its associated factors. Therefore, the findings based on the translated instrument should be interpreted with appropriate caution. Future studies should undertake formal cultural adaptation and psychometric validation of the Hindi RHDS in Indian psychiatric populations. Additionally, assessments relied partly on patient self-report and were not blinded, which may introduce reporting and observer bias. There may also be construct overlap between some discharge-planning variables and the knowledge-related items of the RHDS, as discharge education and communication of discharge-related information may directly contribute to patients’ perceived knowledge and preparedness. Therefore, associations between discharge-planning variables and RHDS scores should be interpreted cautiously and should not necessarily be considered evidence of independent effects. Some potentially relevant variables, such as caregiver burden, health literacy, and quality of discharge counselling, were not systematically assessed and may have influenced the findings. Finally, discharge readiness was measured at a single time point without follow-up, limiting conclusions about its impact on post-discharge outcomes such as adherence or readmission.

In summary, the study demonstrates that although most psychiatric inpatients report moderate readiness for discharge, significant deficiencies remain in knowledge, structured education, and diagnosis-sensitive interventions. Strengthening discharge planning processes, standardising patient and caregiver education, and adopting high-risk discharge pathways for schizophrenia may help reduce relapse, improve continuity of care, and enhance long-term outcomes. Based on the findings of this study, the authors recommend that future multicentric longitudinal follow-up studies are warranted to evaluate the impact of discharge readiness on long-term outcomes, including treatment adherence, functional recovery, quality of life, and readmission rates.

## Conclusions

In comparison to the maximum achievable readiness scores, the findings of this study indicate that there remains substantial scope for strengthening discharge preparedness among psychiatric inpatients. While most patients reported being physically and emotionally stable at the time of discharge, significant deficits were noted in the Knowledge domain of the RHDS. These gaps reflect limited understanding of illness, treatment plans, medication management, relapse warning signs, and follow-up requirements - elements that are critical for safe and effective community reintegration.

The results highlight the need to enhance psychoeducation throughout the inpatient stay, with particular emphasis on illness awareness, medication adherence, coping strategies, and crisis management. Implementing structured, standardised, and written discharge protocols can help ensure consistent communication and improve patient comprehension. Providing clear information about nearby mental-health establishments, available community resources, and legal rights under the Mental Healthcare Act (2017) is especially important in the Indian context, where access pathways are often unclear and families shoulder major caregiving responsibilities.

The inclusion of trained social workers or case managers in discharge planning may further strengthen transitions by ensuring appropriate referrals, coordinating follow-up services, and addressing psychosocial needs such as employment, disability certification, and community linkage. Tailored discharge planning, particularly for patients with schizophrenia who showed the lowest readiness, can contribute to better long-term outcomes and reduce preventable readmissions.

Improving the quality of discharge planning may potentially enhance treatment continuity, support recovery, reduce caregiver burden, and contribute to more sustainable use of mental health resources. These potential benefits should be evaluated in future prospective intervention studies.

## Appendices

**Table 5:** Hindi translation of the Readiness for Hospital Discharge Scale

Sl no	Item	Scoring																															
1.	आप घर जाने के लिए शारीरिक रूप से कितने तैयार हैं?	0	1			2			3			4			5			6		7			8		9			10					
		तैयार नहीं है पूरी तरह से तैयार																															
2.	आज आप अपने दर्द या परेशानी का वर्णन कैसे करेगें?	0			1			2			3		4			5			6			7				8			9		10		
		कोई दर्द/असुविधा नहीं गंभीर दर्द/असुविधा																															
3.	आज आप अपनी ताकत का वर्णन कैसे करेगें?	0		1			2			3				4			5				6			7			8			9		10	
		कमजोर शक्तिशाली																															
4.	आज आप अपनी शक्ति का वर्णन कैसे करेगें?	0		1			2			3				4			5				6			7			8			9			10
		कम ऊर्जा अधिक ऊर्जा																															
5.	आज आप घर जाने के लिए भावनात्मक रूप से कितने तैयार हैं?	0		1			2			3				4			5				6			7			8			9			10
		तैयार नहीं है पूरी तरह से तैयार																															
6.	आज आप स्वयं की देखभाल करने की अपनी शारीरिक क्षमता का वर्णन कैसे करेगें? (उदाहरण के लिए, स्वच्छता, चलना, शौच)?	0		1			2			3				4			5				6			7			8			9			10
		सक्षम नहीं पूरी तरह से सक्षम																															
7.	घर जाने के बाद अपनी देखभाल के बारे में आप कितना जानते हैं?	0		1			2			3				4			5				6			7			8			9			10
		कुछ भी नहीं पता सब कुछ पता है																															
8.	घर जाने के बाद अपनी व्यक्तिगत आवश्यकताओं (उदाहरण के लिए, स्वच्छता, स्नान, शौचालय, भोजन) का ध्यान रखने के बारे में आप कितना जानते हैं?	0		1			2			3				4			5				6			7			8			9			10
		कुछ भी नहीं पता सब कुछ पता है																															
9.	घर जाने के बाद अपने इलाज की जरूरतों (उपचार, दवाइयां) का ध्यान रखने के बारे में आप कितना जानते हैं?	0		1			2			3				4			5				6			7			8			9			10
		कुछ भी नहीं पता सब कुछ पता है																															
10	घर जाने के बाद किन समस्याओं पर ध्यान देना है, इसके बारे में आप कितना जानते हैं?	0		1			2			3				4			5				6			7			8			9			10
		कुछ भी नहीं पता सब कुछ पता है																															
11	आपको इस बारे में कितना पता है कि घर जाने के बाद यदि आपको कोई समस्या हो तो किसे और कब फोन करना चाहिए?	0		1			2			3				4			5				6			7			8			9			10
		कुछ भी नहीं पता सब कुछ पता है																															
12	घर जाने के बाद आपको किन चीज़ों का परहेज़ करना है (आपको क्या करने की अनुमति है और क्या नहीं) इस बारे में आपको कितना पता है?	0		1			2			3				4			5				6			7			8			9			10
		कुछ भी नहीं पता सब कुछ पता है																															
13	घर जाने के बाद आगे उपचार में क्या होता है, इसके बारे में आप कितना जानते हैं?	0		1			2			3				4			5				6			7			8			9			10
		कुछ भी नहीं पता सब कुछ पता है																															
14	घर जाने के बाद आप अपने समुदाय में उपलब्ध सेवाओं और जानकारी के बारे में कितना जानते हैं?	0		1			2			3				4			5				6			7			8			9			10
		कुछ भी नहीं पता सब कुछ पता है																															
15	आप घर पर अपनी नीजी ज़िंदगी की ज़रूरतों को कितनी अच्छी तरह से पूरा कर पाएंगे?	0		1			2			3				4			5				6			7			8			9			10
		बिलकुल नहीं बहुत अच्छे से																															
16	आप घर पर अपनी व्यक्तिगत देखभाल (उदाहरण के लिए, स्वच्छता, स्नान, शौच, भोजन) कितनी अच्छी तरह से कर पाएंगे?	0		1			2			3				4			5				6			7			8			9			10
		बिलकुल नहीं बहुत अच्छे से																															
17	आप अपने उपचार (उदाहरण के लिए, शल्य चिकित्सा चीरे की देखभाल, सांस की कसरत, अन्‍य कसरत, पुनर्वास, या सही मात्रा में और सही समय पर अपनी दवाइयां लेना) घर पर कितनी अच्छी तरह से कर पाएंगे?	0		1			2			3				4			5				6			7			8			9			10
		बिलकुल नहीं बहुत अच्छे से																															
18	घर जाने के बाद आपको कितना भावनात्मक सहारा मिलेगा?	0		1			2			3				4			5				6			7			8			9			10
		बिलकुल नहीं बहुत अच्छे से																															
19	घर जाने के बाद यदि आपको व्यक्तिगत देखभाल के लिए आवश्यकता होगी तो आपको कितनी सहायता मिलेगी?	0		1			2			3				4			5				6			7			8			9			10
		बिलकुल नहीं बहुत																															
20	घर जाने के बाद घरेलू गतिविधियों (उदाहरण के लिए, खाना पकाना, सफाई करना, खरीदारी करना, बच्चों की देखभाल करना) में यदि आपको आवश्यकता होगी तो आपको कितनी मदद मिलेगी?	0		1			2			3				4			5				6			7			8			9			10
		बिलकुल नहीं बहुत																															
21	घर जाने के बाद यदि आपको चिकित्सा से संबंधित देखभाल की आवश्यकता होगी (उपचार, दवाइयां) तो आपको कितनी सहायता मिलेगी?	0		1			2			3				4			5				6			7			8			9			10
		बिलकुल नहीं बहुत																															

**Table 6:** Readiness for Hospital Discharge Study – Adult Form

Item	Scoring
1. How physically ready are you to go home?	0 1 2 3 4 5 6 7 8 9 10
	Not ready Totally ready
2. How would you describe your pain or discomfort today?	0 1 2 3 4 5 6 7 8 9 10
	No pain/ discomfort Severe pain/ discomfort
3. How would you describe your strength today?	0 1 2 3 4 5 6 7 8 9 10
	Weak Strong
4. How would you describe your energy today?	0 1 2 3 4 5 6 7 8 9 10
	Low energy High energy
5. How emotionally ready are you to go home today?	0 1 2 3 4 5 6 7 8 9 10
	Not ready Totally ready
6. How would you describe your physical ability to care for yourself today (for example, hygiene, walking, toileting)?	0 1 2 3 4 5 6 7 8 9 10
	Not able Totally able
7. How much do you know about caring for yourself after you go home?	0 1 2 3 4 5 6 7 8 9 10
	Know nothing at all Know all
8. How much do you know about taking care of your personal needs (for example, hygiene, bathing, toileting, eating) after you go home?	0 1 2 3 4 5 6 7 8 9 10
	Know nothing at all Know all
9. How much do you know about taking care of your medical needs (treatments, medications) after you go home?	0 1 2 3 4 5 6 7 8 9 10
	Know nothing at all Know all
10. How much do you know about problems to watch for after you go home?	0 1 2 3 4 5 6 7 8 9 10
	Know nothing at all Know all
11. How much do you know about who and when to call if you have problems after you go home?	0 1 2 3 4 5 6 7 8 9 10
	Know nothing at all Know all
12. How much do you know about restrictions (what you are allowed and not allowed to do) after you go home?	0 1 2 3 4 5 6 7 8 9 10
	Know nothing at all Know all
13. How much do you know about what happens next in your follow-up medical treatment plan after you go home?	0 1 2 3 4 5 6 7 8 9 10
	Know nothing at all Know all
14. How much do you know about services and information available to you in your community after you go home?	0 1 2 3 4 5 6 7 8 9 10
	Know nothing at all Know all
15. How well will you be able to handle the demands of life at home?	0 1 2 3 4 5 6 7 8 9 10
	Not at all Extremely well
16. How well will you be able to perform your personal care (for example, hygiene, bathing, toileting, eating) at home?	0 1 2 3 4 5 6 7 8 9 10
	Not at all Extremely well
17. How well will you be able to perform your medical treatments (for example, caring for a surgical incision, respiratory treatments, exercise, rehabilitation, or taking your medications in the correct amounts and at the correct times) at home?	0 1 2 3 4 5 6 7 8 9 10
	Not at all Extremely well
18. How much emotional support will you have after you go home?	0 1 2 3 4 5 6 7 8 9 10
	None A great deal
19. How much help will you have if needed with your personal care after you go home?	0 1 2 3 4 5 6 7 8 9 10
	None A great deal
20. How much help will you have if needed with household activities (for example, cooking, cleaning, shopping, babysitting) after you go home?	0 1 2 3 4 5 6 7 8 9 10
	None A great deal
21. How much help will you have if needed with your medical care needs (treatments, medications) after you go home?	0 1 2 3 4 5 6 7 8 9 10
	None A great deal
